# Synchronization in Fractional-Order Complex-Valued Delayed Neural Networks

**DOI:** 10.3390/e20010054

**Published:** 2018-01-12

**Authors:** Weiwei Zhang, Jinde Cao, Dingyuan Chen, Fuad E. Alsaadi

**Affiliations:** 1 School of Mathematics and Computational Science, Anqing Normal University, Anqing 246011, China; 2 School of Mathematics, Southeast University, Nanjing 210096, China; 3 Department of Mathematics, Faculty of Science, King Abdulaziz University, Jeddah 21589, Saudi Arabia; 4 Department of Electrical and Computer Engineering, Faculty of Engineering, King Abdulaziz University, Jeddah 21589, Saudi Arabia

**Keywords:** synchronization, comparison theorem, time-delay, fractional order, complex valued neural networks

## Abstract

This paper discusses the synchronization of fractional order complex valued neural networks (FOCVNN) at the presence of time delay. Synchronization criterions are achieved through the employment of a linear feedback control and comparison theorem of fractional order linear systems with delay. Feasibility and effectiveness of the proposed system are validated through numerical simulations.

## 1. Introduction

Recently, complex valued neural networks (CVNN) have attracted more attention in many research fields, such as signal processing, quantum waves, speech synthesis, and so on [1,2,3,4,5]. Unlike real valued neural networks (RVNN), the state vectors, including weights of connections and activation functions of CVNN, derives from the complex valued field. CVNN can help solve some real-world problems that RVNN can never solve. For example, the Exclusive-OR (XOR) problem and the detection of symmetry problem can be solved with a single complex valued neuron with the orthogonal boundaries, whereas neither of them could be achieved by RVNN with such a simple network structure [6]. Generally speaking, the CVNN have more complicated properties and dynamical behaviors [7,8,9]. In fact, the activation functions in RVNN are employed to be bounded and smooth. However, based on Liouville’s theorem [10], a function that is bounded and analytic at the same time in the complex domain must be constant. Therefore, careful selection of the activation functions of CVNN is a challenging task [11]. Hence, considering the dynamical behaviors of CVNN is important and necessary. Existing results have concerned stability and synchronization [12,13,14].

Fractional calculus, which acts with derivatives and integrals of arbitrary order, was firstly proposed by Leibniz in 1695 [15]. Compared with an integer-order model, fractional order models can offer more accurate instrument for memory description and inherited properties of several processes. Some researchers introduced the fractional order derivatives into neural networks; the fractional order neural networks were designed for precisely modelling in real world [16,17,18,19].

It is worth pointing out that the interesting results of integer order CVNN can not be directly extended to fractional-order complex valued neural networks (FOCVNN). The stability and synchronization analysis of fractional order systems, including FOCVNN, are very difficult. Since calculating the fractional order derivatives of Lyapunov functions is complicated, the stability analysis methods for integer order systems such as Lyapunov functional method can not be easily generalized to fractional order systems. Taking these factors into consideration, many researchers have studied the dynamic behaviors of FOCVNN [20,21,22,23,24,25].

Due to finite switching speeds of amplifiers, it is quite difficult to avoid time delays in neural networks. This may induce oscillation and instability behaviors [26,27,28]. Some interesting results have been presented on the stability of FOCVNN with time delay. For instance, in [21], Gronwall inequality, Cauchy-Schiwartz inequality and inequality skills were utilized to consider stability of FOCVNN at the presence of time delay. Existence and uniform stability analysis of FOCVNN with time delays were studied in [22]. Stability analysis of fractional order complex valued neural networks and memristive neural networks with time delays were studied in [23,24]. To the best of our knowledge, only a few research works have considered the synchronization of FOCVNN with time delay. For example, in [25], synchronization of FOCVNN with time delay was achieved by employing linear delay feedback and a fractional order inequality.

Because the fractional order systems cannot have any exact non-constant periodic solution [29,30], we consider in this paper, from a numerical point of view, that a periodic solution is an extremely-near periodic trajectory. The main goal of this paper is to study the synchronization of FOCVNN with time delay by adopting a new strategy, and some interesting results are obtained. To ensure synchronization, sufficient conditions are established by constructing a Lyapunov function, employing a fractional order inequality and comparison theorem of fractional order linear systems when there is a time delay.

## 2. Preliminaries and Model Description

The literature gives some definitions of the fractional order derivatives, including the Riemann-Liouville definition and Caputo definition. The Caputo derivative only requires initial conditions given in terms of integer-order derivatives, thus it is more applicable in the real world. Therefore, this paper considers the Caputo derivative.

**Definition** **1** ([31]). *The Caputo derivative of fractional order α of a function φ(t) is defined by:*

$$D^{\alpha }\varphi \left(t\right)=\frac{1}{\Gamma (m-\alpha )}\int_{t_{0}}^{t}{\left(t-\tau \right)}^{m-\alpha -1}\varphi^{\left(m\right)}\left(\tau \right)d\tau ,$$

*where $t\geq t_{0}$, $m-1<\alpha <m\in Z^{+}$, Γ(·) is the Gamma function, $\Gamma \left(s\right)=\int_{0}^{\infty }t^{s-1}e^{-t}dt$.*

This paper proposes a class of FOCVNN at the presence of time delay as master system, which is expressed as:

(1) $$D^{\alpha }z_{j}\left(t\right)=-c_{j}z_{j}\left(t\right)+\sum_{k=1}^{n}a_{jk}f_{k}\left(z_{k}\left(t\right)\right)+\sum_{k=1}^{n}b_{jk}g_{k}\left(z_{k}\left(t-\tau \right)\right)+I_{j},$$

or equivalently

(2) $$D^{\alpha }z\left(t\right)=-Cz\left(t\right)+Af\left(z\left(t\right)\right)+g\left(z\left(t-\tau \right)\right)+I\left(t\right),$$

where 0<α<1,j=1,2,⋯,n, *n* is the number of units in a neural networks, $z_{j}\left(t\right)$ corresponds to the state of the *j*-th unit at time *t*, denotes $z\left(t\right)={\left(z_{1}\left(t\right),\cdots ,z_{n}\left(t\right)\right)}^{T}\in C^{n}$, $C=diag\left(c_{1},\cdots ,c_{n}\right)\in R^{n\times n}$ with $c_{j}>0$ is the self-regulating parameters of the neurons. $I\left(t\right)={\left(I_{1}\left(t\right),I_{2}\left(t\right),\cdots ,I_{n}\left(t\right)\right)}^{T}\in C^{n}$ represents the external input, $A={\left(a_{jk}\right)}_{n\times n}$ and $B={\left(b_{jk}\right)}_{n\times n}$ are the connective weights matrix in the presence and absence of delay, respectively. Functions $f_{k}\left(z_{k}\left(t\right)\right):C^{n}\to C^{n}$ and $g_{k}\left(z_{k}\left(t-\tau \right)\right):C^{n}\to C^{n}$ are the complex valued activation functions of the *k*th unit at time *t* and t−τ, respectively, τ>0 is the transmission delay, denotes $f\left(z\left(t\right)\right)={\left(f_{1}\left(z_{1}\left(t\right)\right),\cdots ,f_{n}\left(z_{n}\left(t\right)\right)\right)}^{T}$, $g\left(z\left(t-\tau \right)\right)={\left(g_{1}\left(z_{1}\left(t-\tau \right)\right),\cdots ,g_{n}\left(z_{n}\left(t-\tau \right)\right)\right)}^{T}$.

The slave system is given:

(3) $$D^{\alpha }z_{j}^{\prime }\left(t\right)=-c_{j}z_{j}^{\prime }\left(t\right)+\sum_{k=1}^{n}a_{jk}f_{k}\left(z_{k}^{\prime }\left(t\right)\right)+\sum_{k=1}^{n}b_{jk}g_{k}\left(z_{k}^{\prime }\left(t-\tau \right)\right)+I_{j}+U_{j}\left(t\right),$$

or equivalently

(4) $$D^{\alpha }z^{\prime }\left(t\right)=-Cz^{\prime }\left(t\right)+Af\left(z^{\prime }\left(t\right)\right)+g\left(z^{\prime }\left(t-\tau \right)\right)+I\left(t\right)+U\left(t\right),$$

where $z^{\prime }\left(t\right)={\left(z_{1}^{\prime }\left(t\right),\cdots ,z_{n}^{\prime }\left(t\right)\right)}^{T}\in C^{n}$ is the state vector of the system response, $U\left(t\right)={\left(u_{1}\left(t\right),\cdots ,u_{n}\left(t\right)\right)}^{T}$ is a suitable controller.

In following, some assumptions and useful lemmas are presented to proof the main results.

**Assumption** **1.** *Let z(t)=x(t)+iy(t), f(z(t)) and g(z(t−τ)) are analytic and can be expressed, while separating the real and imaginary parts, as*

$$\begin{array}{c} \begin{array}{c} f\left(z\left(t\right)\right)=f^{R}\left(x\left(t\right),y\left(t\right)\right)+if^{I}\left(x\left(t\right),y\left(t\right)\right), \end{array} \end{array}$$

$$\begin{array}{c} \begin{array}{c} g\left(z\left(t-\tau \right)\right)=g^{R}\left(x\left(t-\tau \right),y\left(t-\tau \right)\right)+ig^{I}\left(x\left(t-\tau \right),y\left(t-\tau \right)\right), \end{array} \end{array}$$

*where $f^{R}\left(\cdot ,\cdot \right)=Re\left(f\left(\cdot ,\cdot \right)\right)={\left(f_{1}^{R}\left(x_{1},y_{1}\right),\cdots ,f_{n}^{R}\left(x_{n},y_{n}\right)\right)}^{T}$, $f^{I}\left(\cdot ,\cdot \right)=Im\left(f\left(\cdot ,\cdot \right)\right)=(f_{1}^{I}\left(x_{1},y_{1}\right),\cdots ,f_{n}^{I}\left(x_{n},y_{n}\right){)}^{T}$, $g^{R}\left(\cdot ,\cdot \right)=Re\left(g\left(\cdot ,\cdot \right)\right)={\left(g_{1}^{R}\left(x_{1},y_{1}\right),\cdots ,g_{n}^{R}\left(x_{n},y_{n}\right)\right)}^{T}$, $g^{I}\left(\cdot ,\cdot \right)=Im\left(g\left(\cdot ,\cdot \right)\right)=(g_{1}^{I}\left(x_{1},y_{1}\right),\cdots ,g_{n}^{I}\left(x_{n},y_{n}\right){)}^{T}$.*

**Assumption** **2.** *The functions $f_{j}^{R}\left(\cdot ,\cdot \right),f_{j}^{I}\left(\cdot ,\cdot \right),g_{j}^{R}\left(\cdot ,\cdot \right),g_{j}^{I}\left(\cdot ,\cdot \right)$ satisfy the following conditions: there exist positive constants $F_{j}^{RR},F_{j}^{RI},F_{j}^{IR},F_{j}^{II},G_{j}^{RR},G_{j}^{RI},G_{j}^{IR},G_{j}^{II},$ such that*

$$\begin{array}{c} \begin{array}{c} |f_{j}^{R}\left(u^{\prime },v^{\prime }\right)-f_{j}^{R}\left(u,v\right)|\leq F_{j}^{RR}|u^{\prime }-u|+F_{j}^{RI}\left|v^{\prime }-v\right|, \end{array} \end{array}$$

$$\begin{array}{c} \begin{array}{c} |f_{j}^{I}\left(u^{\prime },v^{\prime }\right)-f_{j}^{I}\left(u,v\right)|\leq F_{j}^{IR}|u^{\prime }-u|+F_{j}^{II}\left|v^{\prime }-v\right|, \end{array} \end{array}$$

$$\begin{array}{c} \begin{array}{c} |g_{j}^{R}\left(u^{\prime },v^{\prime }\right)-g_{j}^{R}\left(u,v\right)|\leq G_{j}^{RR}|u^{\prime }-u|+G_{j}^{RI}\left|u^{\prime }-u\right|, \end{array} \end{array}$$

$$\begin{array}{c} \begin{array}{c} |g_{j}^{I}\left(u^{\prime },v^{\prime }\right)-g_{j}^{I}\left(u,v\right)|\leq G_{j}^{IR}|u^{\prime }-u|+G_{j}^{II}\left|u^{\prime }-u\right|, \end{array} \end{array}$$

*for all $\left(u,v\right),\left(u^{\prime },v^{\prime }\right)\in R^{2}$.*

Note that Assumption 2 is very important. Compare with the Lipschitz condition $|f_{j}\left(u^{\prime }\right)-f_{j}\left(u\right)|\leq F_{j}\left|u^{\prime }-u\right|$, Assumption 2 is the general Lipschitz condition. In CVNN, the activation functions cannot be bounded and analytic, careful selection of the activation functions of CVNN is a challenge task. Therefore, under Assumption 2, the results in this paper have been obtained.

**Lemma** **1** ([32]). *Suppose $x\left(t\right)\in R^{n}$ is a continuous and differentiable vector-value function. Then, for any time instant $t\geq t_{0}$, we get*

(5) $$D^{\alpha }x^{T}\left(t\right)x\left(t\right)\leq 2x^{T}\left(t\right)D^{\alpha }x\left(t\right),$$

*where 0<α<1.*

**Lemma** **2** ([33]). *Suppose $W\left(t\right)\in R^{1}$ is a continuous differentiable and nonnegative function, which satisfies*

(6) $$\begin{array}{c} \left\{\begin{array}{c} D^{\alpha }W\left(t\right)\leq -aW\left(t\right)+bW\left(t-\tau \right),0<\alpha <1\\ W(t)=\phi (t)\geq 0,t\in [-\tau ,0] \end{array}\right. \end{array}$$

*where t∈[0,+∞). If a>b>0, for all ϕ(t)≥0,τ>0, then $\lim_{t\to +\infty }W\left(t\right)=0$.*

## 3. Main Results

This section derives the synchronization conditions of FOCVNN with time delay by designing a suitable controller.

Assuming that $e\left(t\right)=z^{\prime }\left(t\right)-z\left(t\right)$ is the synchronization error, then the system’s error can be computed as

(7) $$\begin{array}{ccc} D^{\alpha }e_{j}\left(t\right) & = & -c_{j}e_{j}\left(t\right)+\sum_{k=1}^{n}a_{jk}\left[f_{k}\left(z_{k}^{\prime }\left(t\right)\right)-f_{k}\left(z_{k}\left(t\right)\right)\right]\\ & & +\sum_{k=1}^{n}b_{jk}\left[g_{k}\left(z_{j}^{\prime }\left(t-\tau \right)\right)-g_{k}\left(z_{k}\left(t-\tau \right)\right)\right]+U_{j}\left(t\right). \end{array}$$

The vector form as follows:

(8) $$D^{\alpha }e\left(t\right)=-Ce\left(t\right)+A\left[f\left(z^{\prime }\left(t\right)\right)-f\left(z\left(t\right)\right)\right]+B\left[g\left(z^{\prime }\left(t-\tau \right)\right)-g\left(z\left(t-\tau \right)\right)\right]+U\left(t\right).$$

In the following, the notations are used:

(9) $$\begin{array}{c} z\left(t\right)=x\left(t\right)+iy\left(t\right),z^{\prime }\left(t\right)=x^{\prime }\left(t\right)+iy^{\prime }\left(t\right), \end{array}$$

(10) $$\begin{array}{c} e^{R}\left(t\right)=x^{\prime }\left(t\right)-x\left(t\right),e^{I}\left(t\right)=y^{\prime }\left(t\right)-y\left(t\right). \end{array}$$

Select the control input function U(t)=u(t)+iv(t) as the following form:

(11) $$u\left(t\right)=\eta \left(x^{\prime }\left(t\right)-x\left(t\right)\right),v\left(t\right)=\eta^{\prime }\left(y^{\prime }\left(t\right)-y\left(t\right)\right),$$

where each $\eta =diag\left(\eta_{1},\cdots ,\eta_{n}\right),\eta^{\prime }=diag\left(\eta_{1}^{\prime },\cdots ,\eta_{n}^{\prime }\right)$ with $\eta_{j}>0,\eta_{j}^{\prime }>0\;\left(i=1,\cdots ,n\right)$ denote the control gain.

Then the system’s error can be given as

(12) $$\begin{array}{ccc} D^{\alpha }e^{R}\left(t\right) & = & -\Omega e^{R}\left(t\right)+A^{R}\left[f^{R}\left(x^{\prime }\left(t\right),y^{\prime }\left(t\right)\right)-f^{R}\left(x\left(t\right),y\left(t\right)\right)\right]\\ & & -A^{I}\left[f^{I}\left(x^{\prime }\left(t\right),y^{\prime }\left(t\right)\right)-f^{I}\left(x\left(t\right),y\left(t\right)\right)\right]\\ & & +B^{R}\left[g^{R}\left(x^{\prime }\left(t-\tau \right),y^{\prime }\left(t-\tau \right)\right)-g^{R}\left(x\left(t-\tau \right),y\left(t-\tau \right)\right)\right]\\ & & -B^{I}\left[g^{I}\left(x^{\prime }\left(t-\tau \right),y^{\prime }\left(t-\tau \right)\right)-g^{I}\left(x\left(t-\tau \right),y\left(t-\tau \right)\right)\right], \end{array}$$

(13) $$\begin{array}{ccc} D^{\alpha }e^{I}\left(t\right) & = & -\Omega^{\prime }e^{I}\left(t\right)+A^{I}\left[f^{R}\left(x^{\prime }\left(t\right),y^{\prime }\left(t\right)\right)-f^{R}\left(x\left(t\right),y\left(t\right)\right)\right]\\ & & +A^{R}\left[f^{I}\left(x^{\prime }\left(t\right),y^{\prime }\left(t\right)\right)-f^{I}\left(x\left(t\right),y\left(t\right)\right)\right]\\ & & +B^{I}\left[g^{R}\left(x^{\prime }\left(t-\tau \right),y^{\prime }\left(t-\tau \right)\right)-g^{R}\left(x\left(t-\tau \right),y\left(t-\tau \right)\right)\right]\\ & & +B^{R}\left[g^{I}\left(x^{\prime }\left(t-\tau \right),y^{\prime }\left(t-\tau \right)\right)-g^{I}\left(x\left(t-\tau \right),y\left(t-\tau \right)\right)\right], \end{array}$$

where $A^{R},B^{R}$ are the real parts of matrix A,B, respectively, $A^{I},B^{I}$ are the imaginary parts of matrix A,B, respectively. $\Omega =diag\left(c_{1}+\eta_{1},\cdots ,c_{n}+\eta_{n}\right),\Omega^{\prime }=diag\left(c_{1}+\eta_{1}^{\prime },\cdots ,c_{n}+\eta_{n}^{\prime }\right).$

The error system (12) and (13) can also be rewritten as

(14) $$\begin{array}{ccc} D^{\alpha }e_{j}^{R}\left(t\right) & = & -\left(c_{j}+\eta_{j}\right)e_{j}^{R}\left(t\right)+\sum_{k=1}^{n}a_{jk}^{R}\left[f_{k}^{R}\left(x_{k}^{\prime }\left(t\right),y_{k}^{\prime }\left(t\right)\right)-f_{k}^{R}\left(x_{k}\left(t\right),y_{k}\left(t\right)\right)\right]\\ & & -\sum_{k=1}^{n}a_{jk}^{I}\left[f_{k}^{I}\left(x_{k}^{\prime }\left(t\right),y_{k}^{\prime }\left(t\right)\right)-f_{k}^{I}\left(x_{k}\left(t\right),y_{k}\left(t\right)\right)\right]\\ & & +\sum_{k=1}^{n}b_{jk}^{R}\left[g_{k}^{R}\left(x_{k}^{\prime }\left(t-\tau \right),y_{k}^{\prime }\left(t-\tau \right)\right)-g_{k}^{R}\left(x_{k}\left(t-\tau \right),y_{k}\left(t-\tau \right)\right)\right]\\ & & -\sum_{k=1}^{n}b_{jk}^{I}\left[g_{k}^{I}\left(x_{k}^{\prime }\left(t-\tau \right),y_{k}^{\prime }\left(t-\tau \right)\right)-g_{k}^{I}\left(x_{k}\left(t-\tau \right),y_{k}\left(t-\tau \right)\right)\right], \end{array}$$

(15) $$\begin{array}{ccc} D^{\alpha }e_{j}^{I}\left(t\right) & = & -\left(c_{j}+\eta_{j}^{\prime }\right)e_{j}^{I}\left(t\right)+\sum_{k=1}^{n}a_{jk}^{I}\left[f_{k}^{R}\left(x_{k}^{\prime }\left(t\right),y_{k}^{\prime }\left(t\right)\right)-f_{k}^{R}\left(x_{k}\left(t\right),y_{k}\left(t\right)\right)\right]\\ & & +\sum_{k=1}^{n}a_{jk}^{R}\left[f_{k}^{I}\left(x_{k}^{\prime }\left(t\right),y_{k}^{\prime }\left(t\right)\right)-f_{k}^{I}\left(x_{k}\left(t\right),y_{k}\left(t\right)\right)\right]\\ & & +\sum_{k=1}^{n}b_{jk}^{I}\left[g_{k}^{R}\left(x_{k}^{\prime }\left(t-\tau \right),y_{k}^{\prime }\left(t-\tau \right)\right)-g_{k}^{R}\left(x_{k}\left(t-\tau \right),y_{k}\left(t-\tau \right)\right)\right]\\ & & +\sum_{k=1}^{n}b_{jk}^{R}\left[g_{k}^{I}\left(x_{k}^{\prime }\left(t-\tau \right),y_{k}^{\prime }\left(t-\tau \right)\right)-g_{k}^{I}\left(x_{k}\left(t-\tau \right),y_{k}\left(t-\tau \right)\right)\right]. \end{array}$$

Note $\lambda_{1}=\min_{1\leq j\leq n}[\left(c_{j}+\eta_{j}\right)-\sum_{k=1}^{n}\frac{1}{2}|a_{jk}^{R}|F_{k}^{RR}-\sum_{k=1}^{n}\frac{1}{2}|a_{kj}^{R}|F_{j}^{RR}-\sum_{k=1}^{n}\frac{1}{2}|a_{jk}^{R}|F_{k}^{RI}-\sum_{k=1}^{n}\frac{1}{2}|a_{jk}^{I}|F_{k}^{IR}-\sum_{k=1}^{n}\frac{1}{2}|a_{kj}^{I}|F_{j}^{IR}-\sum_{k=1}^{n}\frac{1}{2}|a_{jk}^{I}|F_{k}^{II}-\sum_{k=1}^{n}\frac{1}{2}|b_{jk}^{R}|G_{k}^{RR}-\sum_{k=1}^{n}\frac{1}{2}|b_{jk}^{R}|G_{k}^{RI}-\sum_{k=1}^{n}\frac{1}{2}|b_{jk}^{I}|G_{k}^{IR}-\sum_{k=1}^{n}\frac{1}{2}|b_{jk}^{I}|G_{k}^{II}-\sum_{k=1}^{n}\frac{1}{2}|a_{kj}^{I}|F_{j}^{RR}-\sum_{k=1}^{n}\frac{1}{2}|a_{kj}^{R}\left|F_{j}^{IR}\right],$ $\lambda_{2}=\min_{1\leq j\leq n}[\left(c_{j}+\eta_{j}^{\prime }\right)-\sum_{k=1}^{n}\frac{1}{2}|a_{kj}^{R}|F_{j}^{RI}-\sum_{k=1}^{n}\frac{1}{2}|a_{kj}^{I}|F_{j}^{II}-\sum_{k=1}^{n}\frac{1}{2}|a_{jk}^{I}|F_{k}^{RR}-\sum_{k=1}^{n}\frac{1}{2}|a_{jk}^{I}|F_{k}^{RI}-\sum_{k=1}^{n}\frac{1}{2}|a_{kj}^{I}|F_{j}^{RI}-\sum_{k=1}^{n}\frac{1}{2}|a_{jk}^{R}|F_{k}^{IR}-\sum_{k=1}^{n}\frac{1}{2}|a_{jk}^{R}|F_{k}^{II}-\sum_{k=1}^{n}\frac{1}{2}|a_{kj}^{R}|F_{j}^{II}-\sum_{k=1}^{n}\frac{1}{2}|b_{jk}^{I}|G_{k}^{RR}-\sum_{k=1}^{n}\frac{1}{2}|b_{jk}^{I}|G_{k}^{RI}-\sum_{k=1}^{n}\frac{1}{2}|b_{jk}^{R}|G_{k}^{IR}-\sum_{k=1}^{n}\frac{1}{2}|b_{jk}^{R}\left|G_{k}^{II}\right],$ $\mu_{1}=\max_{1\leq j\leq n}[\sum_{k=1}^{n}\frac{1}{2}|b_{kj}^{R}|G_{j}^{RR}+\frac{1}{2}|b_{kj}^{I}|G_{j}^{IR}+\sum_{k=1}^{n}\frac{1}{2}|b_{kj}^{R}|G_{j}^{IR}+\sum_{k=1}^{n}\frac{1}{2}|b_{kj}^{I}\left|G_{j}^{RR}\right],$ $\mu_{2}=\max_{1\leq j\leq n}[\sum_{k=1}^{n}\frac{1}{2}|b_{kj}^{R}|G_{j}^{RI}+\sum_{k=1}^{n}\frac{1}{2}|b_{kj}^{I}|G_{j}^{II}+\sum_{k=1}^{n}\frac{1}{2}|b_{kj}^{I}|G_{j}^{RI}+\sum_{k=1}^{n}\frac{1}{2}|b_{kj}^{R}\left|G_{j}^{II}\right].$

**Theorem** **1.** Suppose Assumptions 1 and 2 hold, the control gains $\eta ,\eta^{\prime }$ satisfy λ>μ>0, then the master system (1) and the slave system (3) are globally asymptotically synchronized, where $\lambda =\min\{\lambda_{1},\lambda_{2}\},$$\mu =\max\{\mu_{1},\mu_{2}\}.$

**Proof.**  See the Appendix A.

If the parameters, states and activation functions in systems (1) and (3) are all selected from real valued field, based on Theorem 1, we get $\lambda_{1}=\min_{1\leq j\leq n}[\left(c_{j}+\eta_{j}\right)-\sum_{k=1}^{n}\frac{1}{2}|a_{jk}^{R}|F_{k}^{RR}-\sum_{k=1}^{n}\frac{1}{2}|a_{kj}^{R}|F_{j}^{RR}-\sum_{k=1}^{n}\frac{1}{2}|b_{jk}^{R}\left|G_{k}^{RR}\right],$ $\lambda_{2}=\min_{1\leq j\leq n}c_{j}$, $\mu_{1}=\max_{1\leq j\leq n}\sum_{k=1}^{n}\frac{1}{2}\left|b_{kj}^{R}\right|G_{j}^{RR}.$ $\mu_{2}=0.$ Thus, one can obtain the following corollary. ☐

**Corollary** **1.** Suppose Assumptions 1 and 2 hold, the control gains η satisfy $\lambda_{1}>\mu_{1}>0$, then the master system (1) and the slave system (3) are globally asymptotically synchronized.

**Remark** **1.** Compared with [21], in this paper, the comparison theorem of linear fractional order systems with delay is adopted to achieve the synchronization of FOCVNN with time delay, and the results are presented. The method is new and effective at designing the synchronization of complex valued neural networks.

**Remark** **2.** Lemmas 1 and 2 play important and useful roles for studying synchronization of FOCVNN. The proposed method can be extended to consider the synchronization of fractional order complex valued memristive neural networks at the existence of delays, including fractional order chaotic and hyperchaotic systems.

## 4. Numerical Simulations

The following fractional order complex valued delayed neural networks is considered as the master system:

(16) $$D^{\alpha }z\left(t\right)=-Cz\left(t\right)+Af\left(z\left(t\right)\right)+Bg\left(z\left(t-\tau \right)\right)+I\left(t\right),$$

where $z\left(t\right)={\left(z_{1}\left(t\right),z_{2}\left(t\right)\right)}^{T},$ and $z_{j}\left(t\right)=x_{j}\left(t\right)+iy_{j}\left(t\right),j=1,2,$ α=0.98,τ=1.

$C=\left(\begin{array}{cc} 2.5 & 0\\ 0 & 2 \end{array}\right),$ $A=\left(\begin{array}{cc} 3+i & -2-5i\\ 1+1.5i & 0.5+i \end{array}\right),$ $B=\left(\begin{array}{cc} -1+2i & 1+i\\ -1.5-1.5i & 1.5+5i \end{array}\right),$ $I\left(t\right)={\left(\sin t-2i\cos t,3\cos\left(t+1\right)+i\sin\left(t-1\right)\right)}^{T}$, $f\left(z\left(t\right)\right)={\left(f_{1}\left(z_{1}\left(t\right)\right),f_{2}\left(z_{2}\left(t\right)\right)\right)}^{T},$ $g\left(z\left(t\right)\right)={\left(g_{1}\left(z_{1}\left(t\right)\right),g_{2}\left(z_{2}\left(t\right)\right)\right)}^{T},$ and $f_{j}\left(z_{j}\right)=\frac{1-e^{-x_{j}}}{1+e^{-x_{j}}}+\frac{1}{1+e^{-y_{j}}},$ $g_{j}\left(z_{j}\right)=\frac{1-e^{-y_{j}}}{1+e^{-y_{j}}}+\frac{1}{1+e^{-x_{j}}},$ for j=1,2.

The slave system is given as:

(17) $$D^{\alpha }z^{\prime }\left(t\right)=-Cz^{\prime }\left(t\right)+Af\left(z^{\prime }\left(t\right)\right)+Bg\left(z^{\prime }\left(t-\tau \right)\right)+I\left(t\right)+U\left(t\right),$$

where $z^{\prime }\left(t\right)={\left(z_{1}^{\prime }\left(t\right),z_{2}^{\prime }\left(t\right)\right)}^{T},z_{j}^{\prime }\left(t\right)=x_{j}^{\prime }\left(t\right)+iy_{j}^{\prime }\left(t\right)\left(j=1,2\right),$ $U\left(t\right)={\left(U_{1}\left(t\right),U_{2}\left(t\right)\right)}^{T}$, $U_{j}\left(t\right)=u_{j}\left(t\right)+iv_{j}\left(t\right)\left(j=1,2\right)$ is the control function to be designed later.

**Figure 1:**
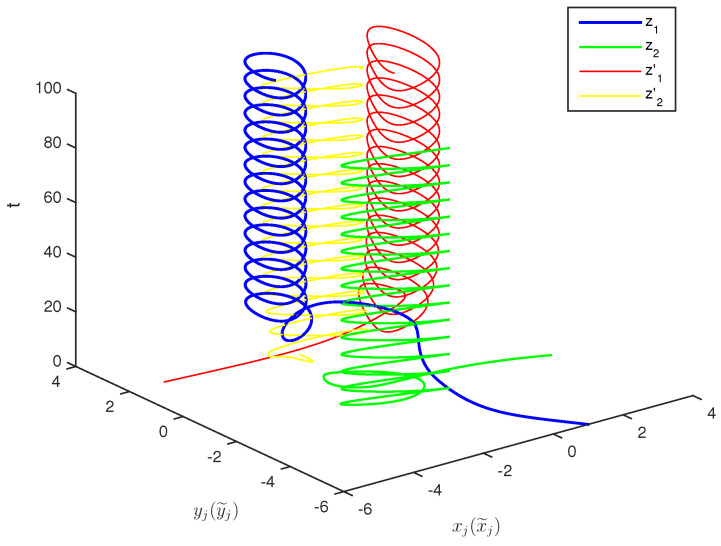
Curves of $z_{1},z_{2},z_{1}^{\prime },z_{2}^{\prime }$ in 3-dimensional space without control.

**Figure 2:**
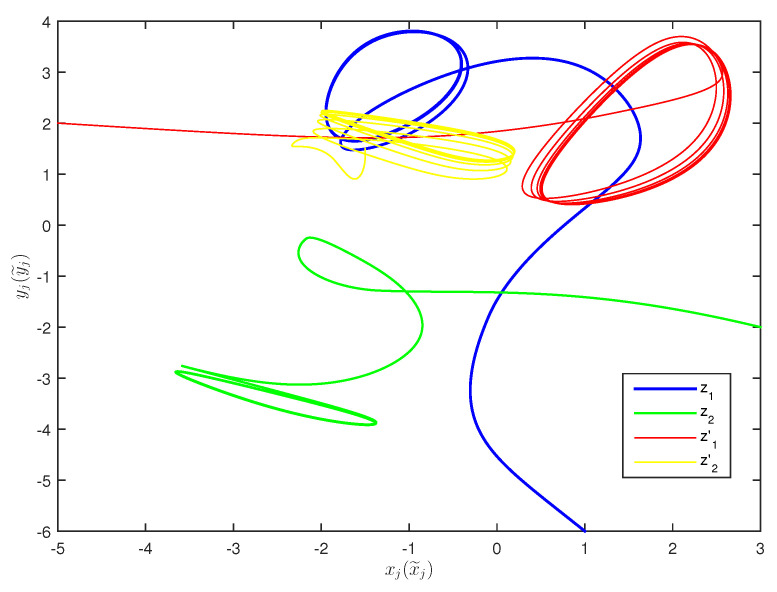
Curves of $z_{1},z_{2},z_{1}^{\prime },z_{2}^{\prime }$ in 2-dimensional space without control.

**Figure 3:**
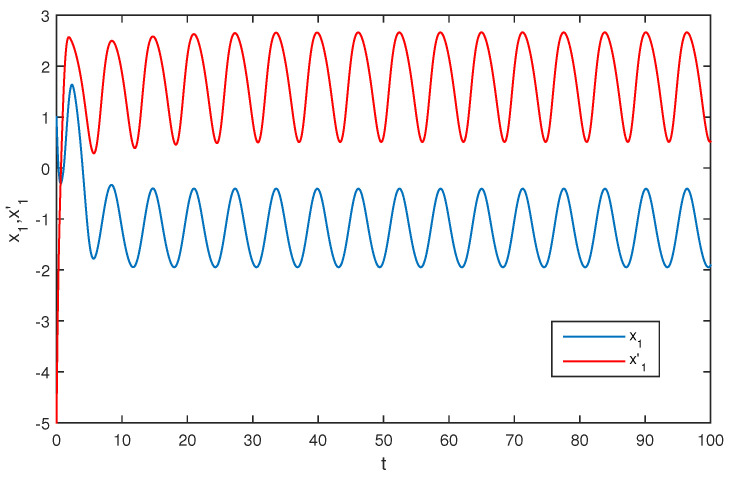
The trajectories of $x_{1},x_{1}^{\prime }$ without control.

**Figure 4:**
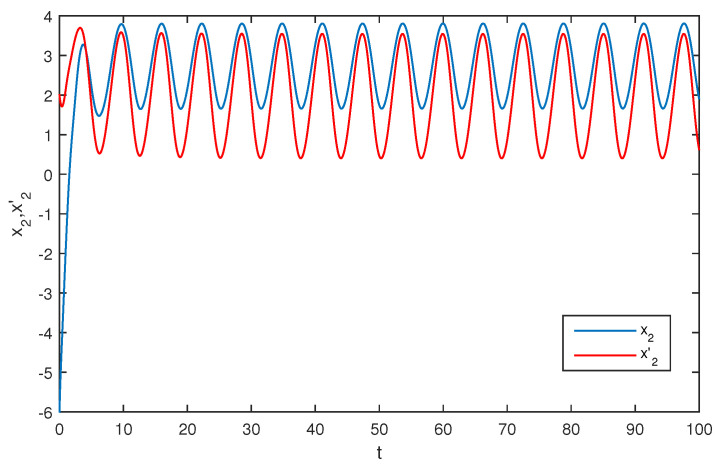
The trajectories of $x_{2},x_{2}^{\prime }$ without control.

**Figure 5:**
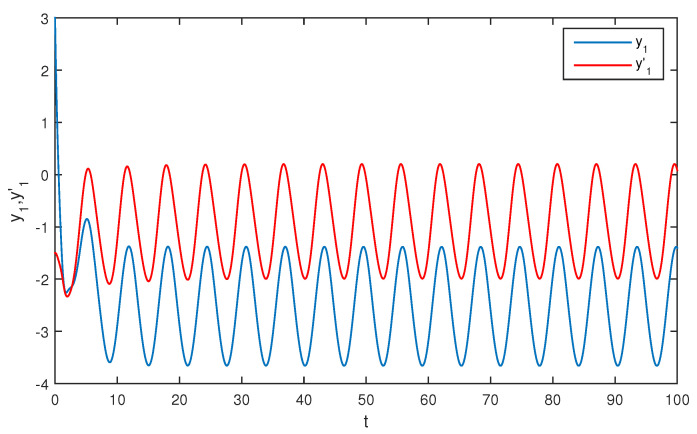
The trajectories of $y_{1},y_{1}^{\prime }$ without control.

**Figure 6:**
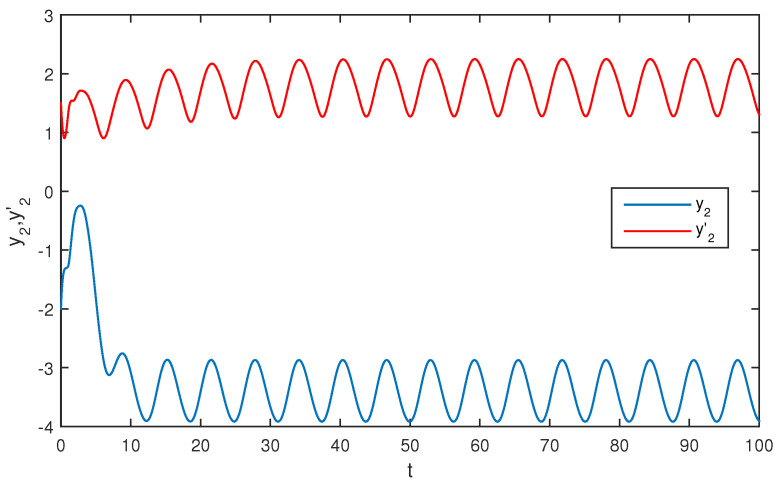
The trajectories of $y_{2},y_{2}^{\prime }$ without control.

The initial values are selected $z_{1}\left(s\right)=1-2i,z_{2}\left(s\right)=2-4i,z_{1}^{\prime }\left(s\right)=-1+2i,z_{2}^{\prime }\left(s\right)=-3+3i$ for s∈[−1,0]. The curves of $z_{1}\left(t\right),z_{2}\left(t\right)$ and $z_{1}^{\prime }\left(t\right),z_{2}^{\prime }\left(t\right)$ are shown without controller in Figure 1 and Figure 2. Figure 3, Figure 4, Figure 5 and Figure 6 depict the time evolution of the real and imaginary parts of $z_{1}\left(t\right),z_{2}\left(t\right)$ and $z_{1}^{\prime }\left(t\right),z_{2}^{\prime }\left(t\right)$ with control gains $\eta =\eta^{\prime }=0.$ The simulation results show that the master system cannot synchronize the slave system without a controller.

**Figure 7:**
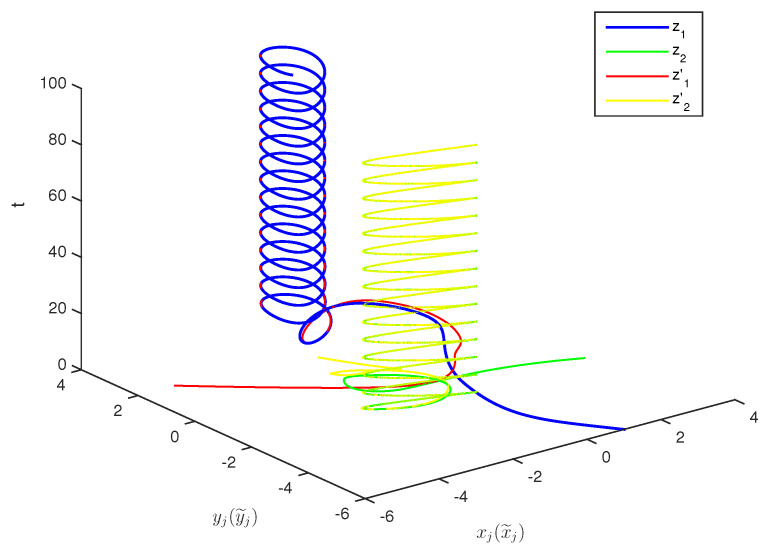
Curves of $z_{1},z_{2},z_{1}^{\prime },z_{2}^{\prime }$ in 3-dimensional space with controller.

**Figure 8:**
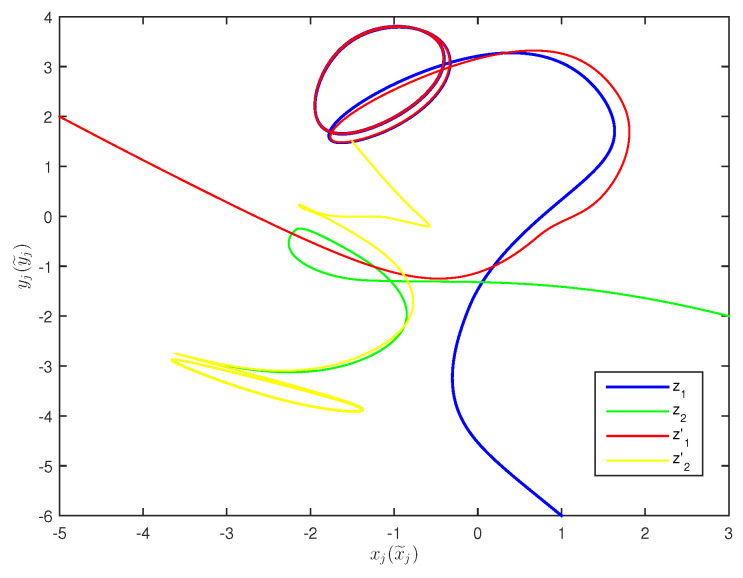
Curves of $z_{1},z_{2},z_{1}^{\prime },z_{2}^{\prime }$ in 2-dimensional space with controller.

**Figure 9:**
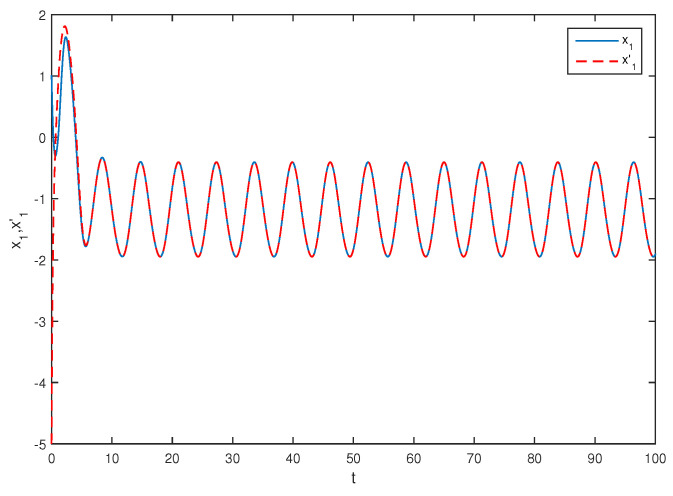
The synchronization trajectories of $x_{1},x_{1}^{\prime }$ with controller.

**Figure 10:**
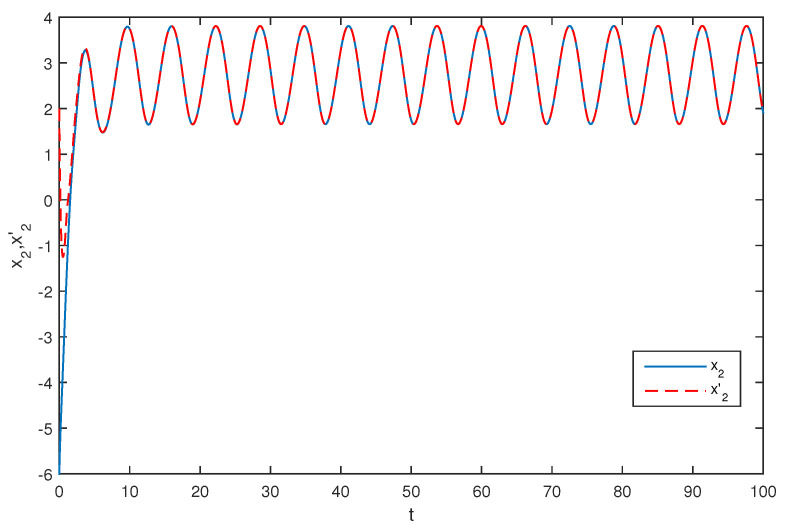
The synchronization trajectories of $x_{2},x_{2}^{\prime }$ with controller.

**Figure 11:**
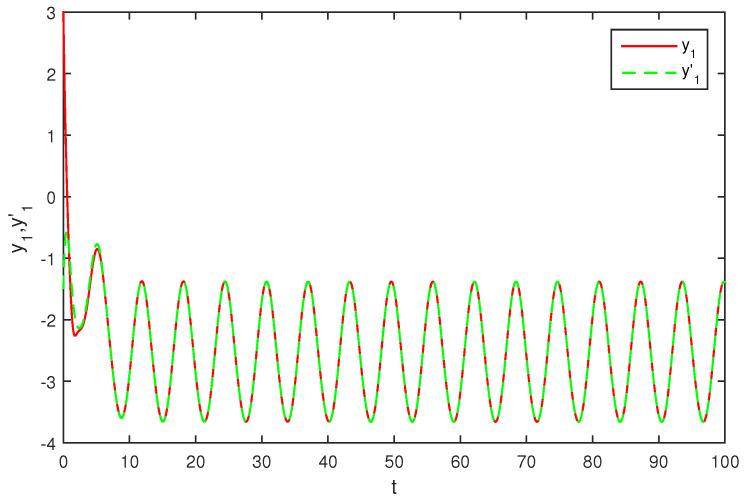
The synchronization trajectories of $y_{1},y_{1}^{\prime }$ with controller.

**Figure 12:**
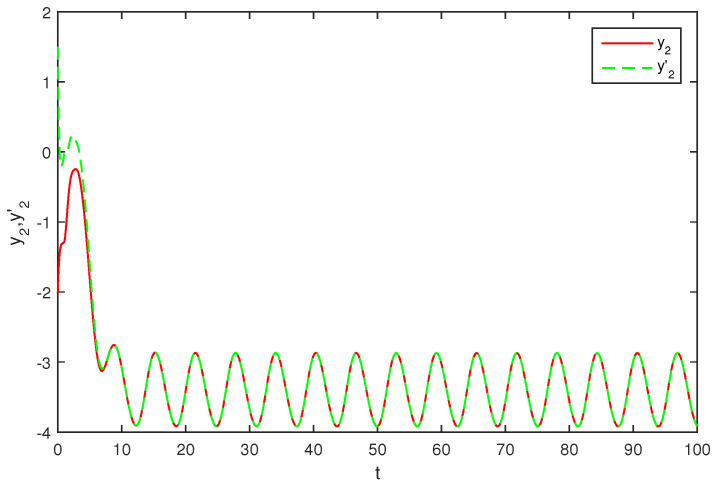
The synchronization trajectories of $y_{2},y_{2}^{\prime }$ with controller.

**Figure 13:**
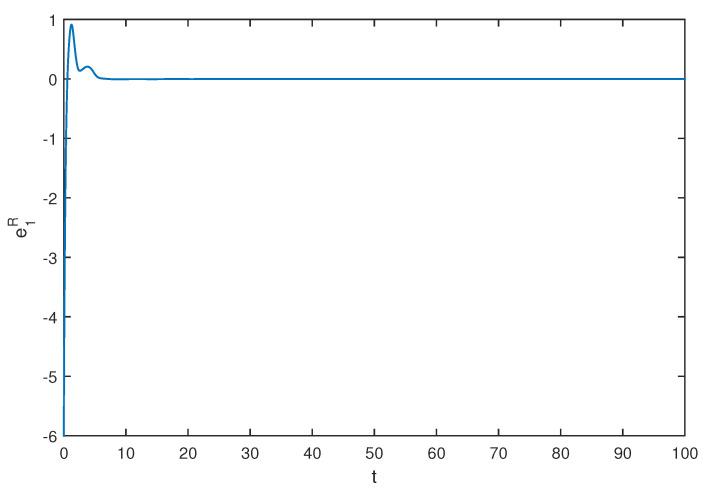
The synchronization error $e_{1}^{R}$ state.

**Figure 14:**
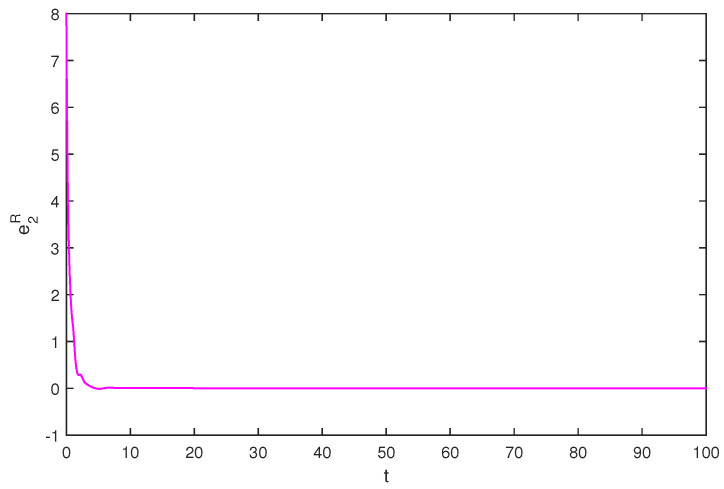
The synchronization error $e_{2}^{R}$ state.

**Figure 15:**
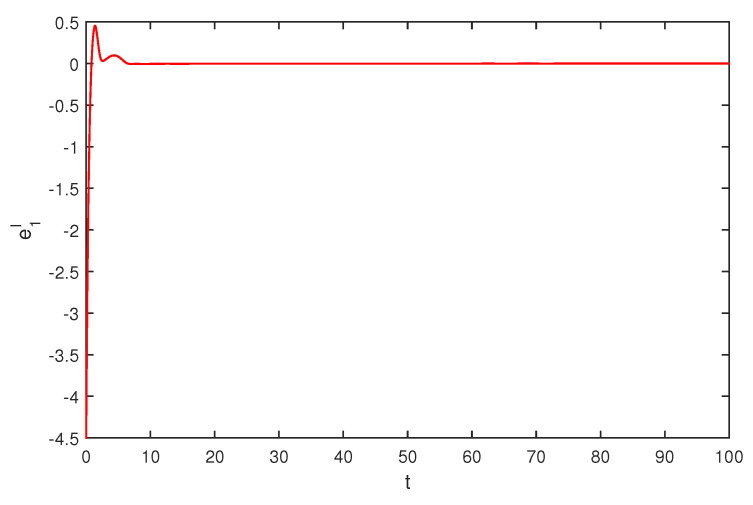
The synchronization error $e_{1}^{I}$ state.

**Figure 16:**
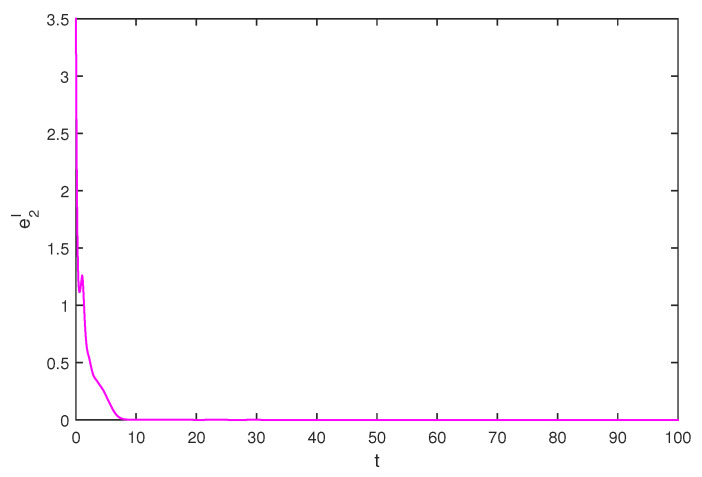
The synchronization error $e_{2}^{I}$ state.

If we select the control gain $\eta_{1}=\eta_{2}=1,\eta_{1}^{\prime }=\eta_{2}^{\prime }=2$, by simple computing, the condition of Theorem 1 is satisfied. The initial values are selected $z_{1}\left(s\right)=1-2i,z_{2}\left(s\right)=2-4i,z_{1}^{\prime }\left(s\right)=-1+2i,z_{2}^{\prime }\left(s\right)=-3+3i,$ for s∈[−1,0]. The curves of $z_{1}\left(t\right),z_{2}\left(t\right)$ and $z_{1}^{\prime }\left(t\right),z_{2}^{\prime }\left(t\right)$ are shown with controller in Figure 7 and Figure 8. The synchronization errors of real and imaginary parts of $z_{1}\left(t\right),z_{2}\left(t\right)$, $z_{1}^{\prime }\left(t\right),z_{2}^{\prime }\left(t\right)$ are shown in Figure 9, Figure 10, Figure 11 and Figure 12, the synchronization trajectories of real and imaginary parts of $z_{1}\left(t\right),z_{2}\left(t\right)$, $z_{1}^{\prime }\left(t\right),z_{2}^{\prime }\left(t\right)$ are shown in Figure 13, Figure 14, Figure 15 and Figure 16, which indicates that the slave system (17) achieved synchronization with the master system (16).

## 5. Conclusions

Compared with real valued neural networks, FOCVNN has more complicated properties and dynamical behaviors. In this paper, the synchronization of FOCVNN with time delay is considered. An error feedback controller is designed by using the comparison theorem of linear fractional order systems with delay and a fractional inequality. An example is proposed to demonstrate the correctness and effectiveness of the obtained results. The method is not only easy to apply for achieving the synchronization of FOCVNN with delay, but also has improved the previous results. The results obtained are still suitable for synchronization of a fractional order real valued neural network with delay. The stability and synchronization of FOCVNN still remain open topics which need to be pursued in the future.

## Abbreviations

The following abbreviations are used in this manuscript:

CVNN	complex valued neural networks
RVNN	real valued neural networks
FOCVNN	fractional-order complex-valued neural networks

## Appendix A

Proof of Theorem 1:

Construct an auxiliary function:

(A1) $$V\left(e\left(t\right)\right)=\frac{1}{2}\sum_{j=1}^{n}{\left(e_{j}^{R}\left(t\right)\right)}^{2}+\frac{1}{2}\sum_{j=1}^{n}{\left(e_{j}^{I}\left(t\right)\right)}^{2}.$$

Using Lemma 1, then

$$\begin{array}{ccc} D^{\alpha }V\left(e\left(t\right)\right) & \leq & e_{j}^{R}\left(t\right)D^{\alpha }\sum_{j=1}^{n}e_{j}^{R}\left(t\right)+e_{j}^{I}\left(t\right)D^{\alpha }\sum_{j=1}^{n}e_{j}^{I}\left(t\right)\\ & = & e_{j}^{R}\left(t\right)\sum_{j=1}^{n}\{-\left(c_{j}+\eta_{j}\right)e_{j}^{R}\left(t\right)+\sum_{k=1}^{n}a_{jk}^{R}\left[f_{k}^{R}\left(x_{k}^{\prime }\left(t\right),y_{k}^{\prime }\left(t\right)\right)-f_{k}^{R}\left(x_{k}\left(t\right),y_{k}\left(t\right)\right)\right]\\ & & -\sum_{k=1}^{n}a_{jk}^{I}\left[f_{k}^{I}\left(x_{k}^{\prime }\left(t\right),y_{k}^{\prime }\left(t\right)\right)-f_{k}^{I}\left(x_{k}\left(t\right),y_{k}\left(t\right)\right)\right]\\ & & +\sum_{k=1}^{n}b_{jk}^{R}\left[g_{k}^{R}\left(x_{k}^{\prime }\left(t-\tau \right),y_{k}^{\prime }\left(t-\tau \right)\right)-g_{k}^{R}\left(x_{k}\left(t-\tau \right),y_{k}\left(t-\tau \right)\right)\right]\\ & & -\sum_{k=1}^{n}b_{jk}^{I}\left[g_{k}^{I}\left(x_{k}^{\prime }\left(t-\tau \right),y_{k}^{\prime }\left(t-\tau \right)\right)-g_{k}^{I}\left(x_{k}\left(t-\tau \right),y_{k}\left(t-\tau \right)\right)\right]\}\\ & & +e_{j}^{I}\left(t\right)\sum_{j=1}^{n}\{-\left(c_{j}+\eta_{j}^{\prime }\right)e_{j}^{I}\left(t\right)+\sum_{k=1}^{n}a_{jk}^{I}\left[f_{k}^{R}\left(x_{k}^{\prime }\left(t\right),y_{k}^{\prime }\left(t\right)\right)-f_{k}^{R}\left(x_{k}\left(t\right),y_{k}\left(t\right)\right)\right]\\ & & +\sum_{k=1}^{n}a_{jk}^{R}\left[f_{k}^{I}\left(x_{k}^{\prime }\left(t\right),y_{k}^{\prime }\left(t\right)\right)-f_{k}^{I}\left(x_{k}\left(t\right),y_{k}\left(t\right)\right)\right]\\ & & +\sum_{k=1}^{n}b_{jk}^{I}\left[g_{k}^{R}\left(x_{k}^{\prime }\left(t-\tau \right),y_{k}^{\prime }\left(t-\tau \right)\right)-g_{k}^{R}\left(x_{k}\left(t-\tau \right),y_{k}\left(t-\tau \right)\right)\right]\\ & & +\sum_{k=1}^{n}b_{jk}^{R}\left[g_{k}^{I}\left(x_{k}^{\prime }\left(t-\tau \right),y_{k}^{\prime }\left(t-\tau \right)\right)-g_{k}^{I}\left(x_{k}\left(t-\tau \right),y_{k}\left(t-\tau \right)\right)\right]\}\\ & \leq & \sum_{j=1}^{n}\{-\left(c_{j}+\eta_{j}\right){\left(e_{j}^{R}\left(t\right)\right)}^{2}+\sum_{k=1}^{n}|e_{j}^{R}\left(t\right)\left|\right|a_{jk}^{R}|[F_{k}^{RR}|e_{k}^{R}\left(t\right)|+F_{k}^{RI}|e_{k}^{I}\left(t\right)\left|\right]\\ & & +\sum_{k=1}^{n}|e_{j}^{R}\left(t\right)||a_{jk}^{I}|[F_{k}^{IR}|e_{k}^{R}\left(t\right)|+F_{k}^{II}|e_{k}^{I}\left(t\right)\left|\right]\\ & & +\sum_{k=1}^{n}|e_{j}^{R}\left(t\right)||b_{jk}^{R}|[G_{k}^{RR}|e_{k}^{R}\left(t-\tau \right)|+G_{k}^{RI}|e_{k}^{I}\left(t-\tau \right)\left|\right]\\ & & +\sum_{k=1}^{n}|e_{j}^{R}\left(t\right)||b_{jk}^{I}|[G_{k}^{IR}|e_{k}^{R}\left(t-\tau \right)|+G_{k}^{II}|e_{k}^{I}\left(t-\tau \right)|]\}\\ & & +\sum_{j=1}^{n}\{-\left(c_{j}+\eta_{j}^{\prime }\right){\left(e_{j}^{I}\left(t\right)\right)}^{2}+\sum_{k=1}^{n}|e_{j}^{I}\left(t\right)\left|\right|a_{jk}^{I}|[F_{k}^{RR}|e_{k}^{R}\left(t\right)|+F_{k}^{RI}|e_{k}^{I}\left(t\right)\left|\right]\\ & & +\sum_{k=1}^{n}|e_{j}^{I}\left(t\right)||a_{jk}^{R}|[F_{k}^{IR}|e_{k}^{R}\left(t\right)|+F_{k}^{II}|e_{k}^{I}\left(t\right)\left|\right]\\ & & +\sum_{k=1}^{n}|e_{j}^{I}\left(t\right)||b_{jk}^{I}|[G_{k}^{RR}|e_{k}^{R}\left(t-\tau \right)|+G_{k}^{RI}|e_{k}^{I}\left(t-\tau \right)\left|\right]\\ & & +\sum_{k=1}^{n}|e_{j}^{I}\left(t\right)||b_{jk}^{R}|[G_{k}^{IR}|e_{k}^{R}\left(t-\tau \right)|+G_{k}^{II}|e_{k}^{I}\left(t-\tau \right)|]\}\\ & \leq & \sum_{j=1}^{n}\{-\left(c_{j}+\eta_{j}\right){\left(e_{j}^{R}\left(t\right)\right)}^{2}+\sum_{k=1}^{n}\frac{1}{2}\left|a_{jk}^{R}\right|F_{k}^{RR}\left[{\left(e_{j}^{R}\left(t\right)\right)}^{2}+{\left(e_{k}^{R}\left(t\right)\right)}^{2}\right]\\ & & +\sum_{k=1}^{n}\frac{1}{2}\left|a_{jk}^{R}\right|F_{k}^{RI}\left[{\left(e_{j}^{R}\left(t\right)\right)}^{2}+{\left(e_{k}^{I}\left(t\right)\right)}^{2}\right]\\ & & +\sum_{k=1}^{n}\frac{1}{2}\left|a_{jk}^{I}\right|F_{k}^{IR}\left[{\left(e_{j}^{R}\left(t\right)\right)}^{2}+{\left(e_{k}^{R}\left(t\right)\right)}^{2}\right]\\ & & +\sum_{k=1}^{n}\frac{1}{2}\left|a_{jk}^{I}\right|F_{k}^{II}\left[{\left(e_{j}^{R}\left(t\right)\right)}^{2}+{\left(e_{k}^{I}\left(t\right)\right)}^{2}\right]\\ & & +\sum_{k=1}^{n}\frac{1}{2}\left|b_{jk}^{R}\right|G_{k}^{RR}\left[{\left(e_{j}^{R}\left(t\right)\right)}^{2}+{\left(e_{k}^{R}\left(t-\tau \right)\right)}^{2}\right]\\ & & +\sum_{k=1}^{n}\frac{1}{2}\left|b_{jk}^{R}\right|G_{k}^{RI}\left[{\left(e_{j}^{R}\left(t\right)\right)}^{2}+{\left(e_{k}^{I}\left(t-\tau \right)\right)}^{2}\right]\\ & & +\sum_{k=1}^{n}\frac{1}{2}\left|b_{jk}^{I}\right|G_{k}^{IR}\left[{\left(e_{j}^{R}\left(t\right)\right)}^{2}+{\left(e_{k}^{R}\left(t-\tau \right)\right)}^{2}\right]\\ & & +\sum_{k=1}^{n}\frac{1}{2}|b_{jk}^{I}\left|G_{k}^{II}\left[{\left(e_{j}^{R}\left(t\right)\right)}^{2}+{\left(e_{k}^{I}\left(t-\tau \right)\right)}^{2}\right]\right\}\\ & & +\sum_{j=1}^{n}\{-\left(c_{j}+\eta_{j}^{\prime }\right){\left(e_{j}^{I}\left(t\right)\right)}^{2}+\sum_{k=1}^{n}\frac{1}{2}\left|a_{jk}^{I}\right|F_{k}^{RR}\left[{\left(e_{j}^{I}\left(t\right)\right)}^{2}+{\left(e_{k}^{R}\left(t\right)\right)}^{2}\right]\\ & & +\sum_{k=1}^{n}\frac{1}{2}\left|a_{jk}^{I}\right|F_{k}^{RI}\left[{\left(e_{j}^{I}\left(t\right)\right)}^{2}+{\left(e_{k}^{I}\left(t\right)\right)}^{2}\right]\\ & & +\sum_{k=1}^{n}\frac{1}{2}\left|a_{jk}^{R}\right|F_{k}^{IR}\left[{\left(e_{j}^{I}\left(t\right)\right)}^{2}+{\left(e_{k}^{R}\left(t\right)\right)}^{2}\right]\\ & & +\sum_{k=1}^{n}\frac{1}{2}\left|a_{jk}^{R}\right|F_{k}^{II}\left[{\left(e_{j}^{I}\left(t\right)\right)}^{2}+{\left(e_{k}^{I}\left(t\right)\right)}^{2}\right]\\ & & +\sum_{k=1}^{n}\frac{1}{2}\left|b_{jk}^{I}\right|G_{k}^{RR}\left[{\left(e_{j}^{I}\left(t\right)\right)}^{2}+{\left(e_{k}^{R}\left(t-\tau \right)\right)}^{2}\right]\\ & & +\sum_{k=1}^{n}\frac{1}{2}\left|b_{jk}^{I}\right|G_{k}^{RI}\left[{\left(e_{j}^{I}\left(t\right)\right)}^{2}+{\left(e_{k}^{I}\left(t-\tau \right)\right)}^{2}\right]\\ & & +\sum_{k=1}^{n}\frac{1}{2}\left|b_{jk}^{R}\right|G_{k}^{IR}\left[{\left(e_{j}^{I}\left(t\right)\right)}^{2}+{\left(e_{k}^{R}\left(t-\tau \right)\right)}^{2}\right]\\ & & +\sum_{k=1}^{n}\frac{1}{2}|b_{jk}^{R}\left|G_{k}^{II}\left[{\left(e_{j}^{I}\left(t\right)\right)}^{2}+{\left(e_{k}^{I}\left(t-\tau \right)\right)}^{2}\right]\right\}\\ & = & -\sum_{j=1}^{n}[\left(c_{j}+\eta_{j}\right)-\sum_{k=1}^{n}\frac{1}{2}|a_{jk}^{R}|F_{k}^{RR}-\sum_{k=1}^{n}\frac{1}{2}|a_{kj}^{R}|F_{j}^{RR}-\sum_{k=1}^{n}\frac{1}{2}\left|a_{jk}^{R}\right|F_{k}^{RI}\\ & & -\sum_{k=1}^{n}\frac{1}{2}|a_{jk}^{I}|F_{k}^{IR}-\sum_{k=1}^{n}\frac{1}{2}|a_{kj}^{I}|F_{j}^{IR}-\sum_{k=1}^{n}\frac{1}{2}|a_{jk}^{I}|F_{k}^{II}-\sum_{k=1}^{n}\frac{1}{2}\left|b_{jk}^{R}\right|G_{k}^{RR}\\ & & -\sum_{k=1}^{n}\frac{1}{2}|b_{jk}^{R}|G_{k}^{RI}-\sum_{k=1}^{n}\frac{1}{2}|b_{jk}^{I}|G_{k}^{IR}-\sum_{k=1}^{n}\frac{1}{2}\left|b_{jk}^{I}\right|G_{k}^{II}\\ & & -\sum_{k=1}^{n}\frac{1}{2}|a_{kj}^{I}|F_{j}^{RR}-\sum_{k=1}^{n}\frac{1}{2}|a_{kj}^{R}\left|F_{j}^{IR}\right]{\left(e_{j}^{R}\left(t\right)\right)}^{2}\\ & & -\sum_{j=1}^{n}[\left(c_{j}+\eta_{j}^{\prime }\right)-\sum_{k=1}^{n}\frac{1}{2}|a_{kj}^{R}|F_{j}^{RI}-\sum_{k=1}^{n}\frac{1}{2}|a_{kj}^{I}|F_{j}^{II}-\sum_{k=1}^{n}\frac{1}{2}\left|a_{jk}^{I}\right|F_{k}^{RR}\\ & & -\sum_{k=1}^{n}\frac{1}{2}|a_{jk}^{I}|F_{k}^{RI}-\sum_{k=1}^{n}\frac{1}{2}|a_{kj}^{I}|F_{j}^{RI}-\sum_{k=1}^{n}\frac{1}{2}\left|a_{jk}^{R}\right|F_{k}^{IR}\\ & & -\sum_{k=1}^{n}\frac{1}{2}|a_{jk}^{R}|F_{k}^{II}-\sum_{k=1}^{n}\frac{1}{2}|a_{kj}^{R}|F_{j}^{II}-\sum_{k=1}^{n}\frac{1}{2}\left|b_{jk}^{I}\right|G_{k}^{RR}\\ & & -\sum_{k=1}^{n}\frac{1}{2}|b_{jk}^{I}|G_{k}^{RI}-\sum_{k=1}^{n}\frac{1}{2}|b_{jk}^{R}|G_{k}^{IR}-\sum_{k=1}^{n}\frac{1}{2}|b_{jk}^{R}\left|G_{k}^{II}\right]{\left(e_{j}^{I}\left(t\right)\right)}^{2}\\ & & +\sum_{j=1}^{n}[\sum_{k=1}^{n}\frac{1}{2}|b_{kj}^{R}|G_{j}^{RR}+\frac{1}{2}\left|b_{kj}^{I}\right|G_{j}^{IR}\\ & & +\sum_{k=1}^{n}\frac{1}{2}|b_{kj}^{R}|G_{j}^{IR}+\sum_{k=1}^{n}\frac{1}{2}|b_{kj}^{I}\left|G_{j}^{RR}\right]{\left(e_{j}^{R}\left(t-\tau \right)\right)}^{2}\\ & & +\sum_{j=1}^{n}[\sum_{k=1}^{n}\frac{1}{2}|b_{kj}^{R}|G_{j}^{RI}+\sum_{k=1}^{n}\frac{1}{2}\left|b_{kj}^{I}\right|G_{j}^{II}\\ & & +\sum_{k=1}^{n}\frac{1}{2}|b_{kj}^{I}|G_{j}^{RI}+\sum_{k=1}^{n}\frac{1}{2}|b_{kj}^{R}\left|G_{j}^{II}\right]{\left(e_{j}^{I}\left(t-\tau \right)\right)}^{2}. \end{array}$$

Then, one gets

(A2) $$\begin{array}{ccc} D^{\alpha }V\left(e\left(t\right)\right) & \leq & -2\lambda_{1}\sum_{j=1}^{n}{\left(e_{j}^{R}\left(t\right)\right)}^{2}-2\lambda_{2}\sum_{j=1}^{n}{\left(e_{j}^{I}\left(t\right)\right)}^{2}\\ & & +2\mu_{1}\sum_{j=1}^{n}{\left(e_{j}^{R}\left(t-\tau \right)\right)}^{2}+2\mu_{2}\sum_{j=1}^{n}{\left(e_{j}^{I}\left(t-\tau \right)\right)}^{2}\\ & \leq & -2\lambda V(e(t))+2\mu V(e(t-\tau )). \end{array}$$

According to Lemma 2, when λ>μ>0, the system (1) synchronizes the system (3).
